# Treatment of Deep Seated Abscesses without External Incision

**Published:** 1885-12

**Authors:** John S. Marshall

**Affiliations:** Chicago, Ill.


ARTICLE VI.

### TREATMENT OF DEEP-SEATED ABSCESSES WITHOUT EXTERNAL INCISION.

BY JOHN S. MARSHALL, M. D., CHICAGO, ILL.

[Read before the Minnesota Dental Society, August 1, 1885.]

By deep-seated abscesses I mean those cases of alveolar abscess which have extended beyond the ordinary limits, and have involved more or less extensively the structures of the jaw, with a tendency to necrosis, or have penetrated the antrum of Highmore or escaped from the neighborhood of the maxilla, and have burrowed downwards between the muscles of the neck, as frequently occurs in abscesses associated with the inferior teeth.

Ordinarily the diagnosis of these cases is not difficult, but occasionally the cause has proved troublesome to find. Abscesses discharging into the antrum, or the nasal fossa, and producing offensive discharges, have been diagnosed as chronic catarrh. One case occuring in the practice of Dr. Edward Maynard, of Washington, D. C., caused by an unerupted inferior wisdom tooth, and discharging into the larynx, setting up an irritative cough with expectoration of pus and mucus, was previously diagnosod by the physicians to be acute bronchitis; others discharging at some point upon the side of the neck have been set down as abscesses originating in the cervical glands, the result of scrofula.

That such abscesses often prove to be serious affections, endangering the health, add sometimes even the life, of the individual, are well established facts.

I purpose, however, in this short paper to confine my remarks to the more common, and, from their location, the more dangerous class of these cases, viz., those originating from disease of the inferior teeth.

The tendency of the suppurative products in these cases is downwards through the external wall of the alveolar process, and to point at the lower margin of the jaw; but it also happens—especially with the molars—that instead of pointing at this location it opens through the internal wall of the alveolar process, and burrows downwards between the muscles of the neck, and may discharge into the throat, or through the external tissues at various points from the submaxillary triangle to the superior border of the clavicle. Any suggestions, therefore, in regard to the treatment of these cases which will tend to cut short the suppurative process, lessen the dangers to health and life, avoid the necessity of operating with the scalpel in a location requiring such delicate dissections and fraught with so much risk to the patient, or to prevent the unsightly and oftimes disgusting scars which follow the external opening of these abscesses, will I think be of interest. The treatment frequently adopted in cases of alveolar abscess is the removal of the cause by the extraction of the offending tooth, trusting to nature to complete the cure.

In extreme cases of this deep-seated variety an incision is made through the external tissues at the lowest point of the abscess, for the purpose of drainage. In those cases, however, where the pus has burrowed deeply into the tissues of the neck it is quite likely that more than one pocket will be formed; consequently the treatment by incision becomes complicated, and sometimes, from the dangers of an extended operation in the superior or inferior carotid triangles, would be precluded altogether.

The surgeon, under such circumstances, has had no alternative but to wait, trusting that the abscess would find an opening for itself at less risk, before the patient should die of pyæmia. The treatment which is suggested comes to our relief in this emegency, and from past experience I am prepared to say, at least, that the duration of these cases can be materially shortened, and many of them speedily cured, without resort to any other operative procedure than the extraction of the diseased tooth and the injection of peroxide of hydrogen into the sac..

Ophthalmologists and aurists have found this agent very useful in the treatment of diseases of the eye and ear, with purulent and muco-purulent discharges, and dentists have been signally successful with it in the treatment of pulp chambers with putrid contents, in ordinary alveolar abscesses and in pyorrhea alveolaris. By injecting an abscess of the deep-seated variety with peroxide of hydrogen, introduced through the alveolus of the extracted tooth, the purulent contents can be thoroughly evacuated.

The oxygen is set free on coming in contact with the products of decomposition, which distends the cavity and forces out the pus through the alveolus by mechanical pressure. Two or three injections of from a half a drachm to an ounce, according to the extent of the abscess, may be required to completely remove the purulent matter, and if given opportunity it will search out and purify every hidden receptacle. I have had several opportunities since its introduction to the notice of the dental profession, by Dr. Walter Coffin,of England, at the London International Medical Congress in 1881,* to test its efficiency in this class of cases, and in extensive periosteal inflammations of the jaw.

In one case, a Mercy Hospital patient, Mary N. Irish, aged twenty-four years, was suffering from a deep-seated abscess associated with the right inferior wisdom tooth for several weeks. The patient was confined to her bed for twenty-six days, with pulse ranging from 100 to 116, and temperature from 101° to 104,8°. She was speedily relieved by extracting the tooth and evacuating the pus. The abscess extended down the neck four and a half inches below the margin of the gums, as was ascertained by the probe. The pulse dropped from 104 to 96 and the temperature from 104.8° to 103° within two hours after the operation. A half ounce of the peroxide was ordered to be injected into the

*Transactions of the Seventh International Medical Congress.

abscess every four hours. This was followed by a decrease in the temperature of one degree each day for three days. The patient then refused to submit to the further use of the remedy at that time,as the evolution of the gas, by distending the sac, caused pain. An increase in the pulse rate and an elevation in the temperture immediately followed. On the fourth day afterwards the pulse was 101 and the temperature 104°.

The peroxide was again used as before, and the pulse and temperature again rapidly fell, but through the obstinacy of the patient the treatment could not be carried out with any degree of satisfaction ; still the fact was established that the remedy antisepticized the pus and evacuated the sac, as indicated by the rapid improvement in the symptoms.

Another case was that of a little girl, aged eleven years with an abscess originating from the right inferior first molar and extending into the tissues of the neck, accompanied with extensive swelling and tenderness, but with no acute pain. The swelling of the parts had followed an attack of severe pain in the tooth and jaw, from which she had suffered three weeks previously. For a week the jaws had been closed, and the only food taken each day was a little milk. The child had been confined to bed for a part of the time, and when presented for treatment looked decidedly ill. The tooth was extracted under ether, and the pus cavity found to extend downwards three inches below the margin of the gum. Very little purulent matter followed the extraction of the tooth, but on injection the pocket with peroxide large quantities was evacuated. The injections were continued once daily for six days, when the patient was pronounced cured, all discharge having ceased, and the swelling nearly disappeared, Under the ordinary treatment I should have expected to have seen the trouble continue for a much longer period, and perhaps to have seen the abscess point low down on the neck.

One other case was that of a lad nine years of age, who had received an injury of the inferior jaw by a fall from his bicycle, resulting in an extensive acute periostitis, involving the teeth and jaw from the second temporary molar of the left side to the ramus of the jaw on the right side. All of the teeth between these points were loose; pus exuded from the gums at the necks of the teeth, and I feared extensive necrosis. The treatment adopted was injections of peroxide beneath the gums at all points where pus was found to exude. The condition at the anterior part of the mouth began to improve at once, but opposite the first permanent molar at the lower margin of the jaw it was necessary a few days later to open an abscess which was about to point there. The injections were kept up for two weeks; all discharge had then ceased, and the teeth had become firm.

Dr. Harlan has recently called attention to the use of this agent in purulent conditions effecting the maxillary sinus,* and I would suggest that it will be found equally valuable in the hands of the surgeon in nearly every variety of suppurative inflammation, especially in periostitis, necrosis, and deep-seated abscesses, where there is difficulty in completely evacuating the purulent matter by the ordinary means.—*Dental Cosmos.*

---

*Archives of Dentistry, page 204, May, 1885.

